# Geopolymer Carbon-Based for Ultra-Wideband Absorbent Applications

**DOI:** 10.3390/molecules25184218

**Published:** 2020-09-14

**Authors:** Ioana Nicoleta Vlasceanu, Ameni Gharzouni, Olivier Tantot, Edson Martinod, Sylvie Rossignol

**Affiliations:** 1 IRCER—Institut de Recherche sur les Céramiques, UMR CNRS 7315, 12 Rue Atlantis, 87068 CEDEX Limoges, France; ioana-nicoleta.vlasceanu@unilim.fr (I.N.V.); ameni.gharzouni@unilim.fr (A.G.); 2 XLIM, UMR CNRS 7252, 123 Avenue Albert Thomas, 87060 CEDEX Limoges, France; olivier.tantot@unilim.fr; 3 XLIM, UMR CNRS 7252, 16 Rue Jules Vallès, 19100 Brive La Gaillarde, France; edson.martinod@unilim.fr

**Keywords:** geopolymer, dielectric properties, absorbent, foam, surfactants, pore size

## Abstract

Dimension reduction, cost efficiency, and environmental sustainability are important factors in absorbent designs. Geopolymers represent an eco-friendly and cost-efficient solution for such applications, and the objective of this study is to develop new geopolymer-based composites with tailored dielectric properties. To develop such composites, different formulations based on three types of carbon and various surfactants are tested. The nonionic surfactant is preferred over the anionic surfactant. Dielectric investigations between 2 and 3.3 GHz are performed. The results reveal that the carbon content and its type (origin) have significant effects on the dielectric characteristics and less on the magnetic characteristics. Indeed, an increase in permittivity from 2 to 24 and an increase from 0.09 to 0.6 for loss tangent are shown with changes in the carbon content and type. A permittivity (ε) of 2.27 and loss (tan δ) of 0.19 are obtained for a pore size of 1.6 mm, for the carbon type with the lowest purity, and with a nonionic surfactant. Finally, it is shown that the addition of magnetite has little impact on the overall magnetic properties of the geopolymer.

## 1. Introduction

Recently, many studies on dielectric properties have been performed at different charge rates for various particles (ferrite, carbon black, among others) in polymer matrices and compared to the properties of the matrix alone. They showed the different types of composites that have a high frequency polyurethane matrix (GHz range) [1,2]. For example, in the range 1–40 GHz [3,4], the microwave absorber used is a mixed foam-like material, which is between polyurethane and polystyrene-carbon with neoprene binders, typically used to prepare microwave absorbent foams for anechoic chambers [5,6]. The most used absorbents are mixed carbonyl ions (e.g., carbon black, graphite powder, carbon nanoparticles) [7] and ferrites [8] with polymers, such as plastic or rubber, in sheets or as foam. To improve absorption characteristics, another class of microwave absorbers has been developed, which is a matrix of conductive polymers with different dielectric and magnetic fillers. For excellent dielectric properties and low-density carbon materials in various forms, such as graphite, carbon black, carbon nanotubes, and carbon fibers, microwave absorbers can be used in the preparation of wave-absorbing materials [9]. Many studies have modified carbon material or combined carbon materials with other products, such as biochar [10,11].

As geopolymer materials are eco-friendly and energy efficient, they can be used to reduce pollution. These materials can be defined as amorphous aluminosilicate binders that are synthesized by the activation of an aluminosilicate source by an alkaline solution at atmospheric pressure and a temperature below 100 °C [12]. Such materials are also defined by the accurate control of their porosity, as they can be dense or turned into foams [13,14]. To control the porosity of the foam, the use of a surfactant can be added. Surfactants are foaming agents, which, in small amounts in solution, can accelerate the formation of foam and ensure stability by preventing collapse. Surfactants can be either natural or of synthetic origin, and ionic or nonionic. The chemical nature of the surfactant will indicate the interactions that govern the hydrophobic attractive interactions between hydrocarbons and the steric and electrostatic repulsive interactions between the polar heads [15]. The critical micellar concentration (CMC) is the concentration from which a surfactant self-associates as a micelle, which depends on the chemical nature of the surfactant [16]. The characteristics of surfactants are highly dependent on the nature of the medium and in particular on the presence of an electrolyte. Indeed, depending on the population of counter-ions, the cohesion between molecules is more or less strong, limiting electrostatic repulsion between neighboring polar groups [17]. The counter-ions will adsorb with the surfactant at the liquid/air interface. The increase in the degree of association of the counter-ions makes it possible to decrease the CMC, and thus the value of the surface tension. The CMC value depends on the nature of the counter ion and decreases in the following order: Li^+^ > Na^+^ > K^+^ > Cs [18].

In this study, new geopolymer compositions based on various types of carbon and surfactants, with tailored dielectric properties, for absorbents are developed. The influence of the type and concentration of the surfactant on the geopolymer, as well as its carbon nature and content, are studied with respect to the dielectric and magnetic properties.

## 2. Results

### 2.1. Surfactant Impact

**Figure 1:**
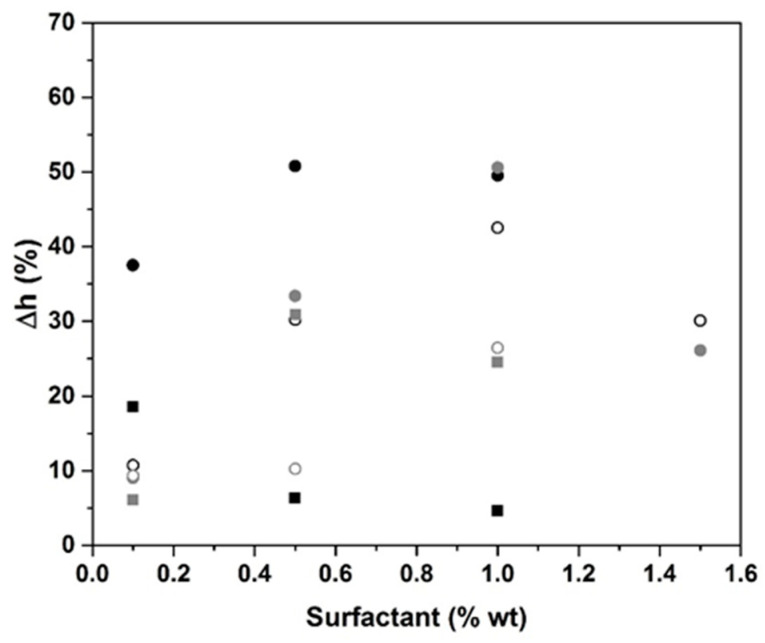
The evolution of volume expansion in the function of the weight percentage of various surfactants for biochar (GB) samples with 3% of carbon (non-ionic: ● BG; ● CG; ○ APG; ○ LQ; anionic: ■ Tego; ■ H66).

Figure 1 shows the volume expansion of the cured samples with 3% biochar (GB) and various types and proportions of surfactant (0.1–1.5 wt.%). Two types of surfactant are used: nonionic (BG, CG, APG, and LQ) and anionic (H66 and Tego). The values of Δh (calculated with Equation (1) presented in the Technical characterization section) vary depending on the type of surfactant. The use of the BG surfactant induces an increase in Δh by 50%, and afterwards, a decrease is observed. This phenomenon is related to the variation in the interfacial strength reaching a maximum, which corresponds to the CMC [19]. With the CG or APG surfactants, the upper limit is reached at 1% (Δh = 50%) [20]. The CMC for BG is at 0.5% addition of surfactant, whereas for CG, APG, and 80-LQ, it is at 1% addition of surfactant. When the surfactant content increases, first, there is a formation of aggregates, which, at a high concentration, lead to the formation of a layer over the surface [21]. The same behavior is observed in the presence of anionic surfactants. A maximum of Δh = 19 and 31% is reached for 0.1% and 0.5% of Tego and H66, respectively. The CMC for anionic surfactants is at 0.5% for H66 and at 0.1% for Tego. Nevertheless, this maximum is weaker than for the anionic surfactant. In fact, the reduction in the electrostatic repulsion between the ionic surfactant head groups in the mixed micelle, owing to the insertion of nonionic hydrophilic groups between these charged groups, is the cause of enhanced micelle formation [22]. Thus, the surfactant CMC and type control the volume expansion. On one hand, the variation of the physicochemical properties of the surfactant above and below the CMC influences the foam stability. Indeed, around the CMC, the surfactant molecules adsorb at the interface of the bubbles to decrease the surface tension, which promotes the foaming and thus the foam expansion [23,24,25]. Above the CMC and for higher concentrations, the surface tension remains low, but the addition of surfactant increases the viscosity of the mixture, which inhibits the foaming and explains the decrease of the foam expansion. In the case of geopolymer, where several ions are in solution, non-ionic surfactants have a more pronounced effect owing to their hydrophilic groups without electric charges [26].

**Figure 2:**
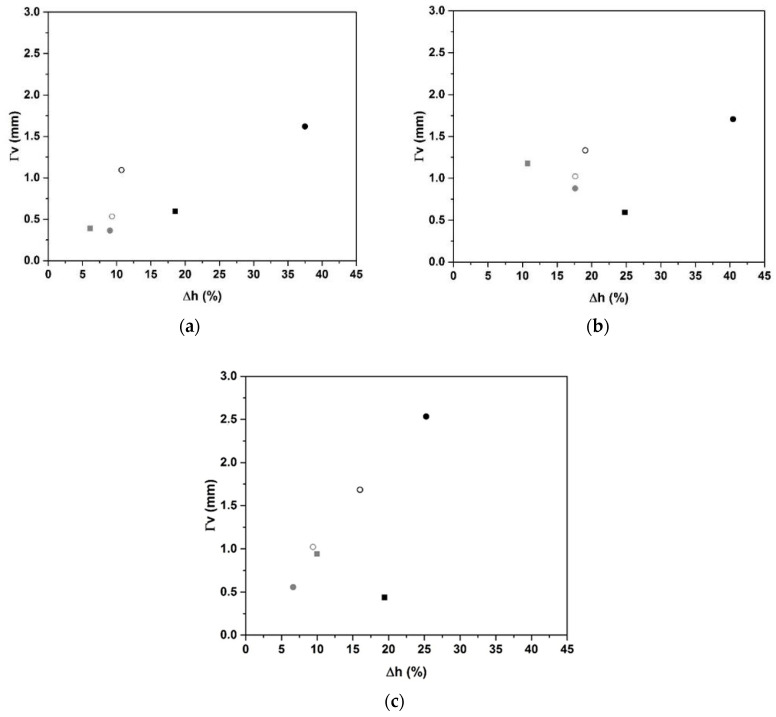
Pore size function of volume expansion of 0.1% addition of various surfactants for GB (**a**), GG^75^ (**b**), and GG^99^ (**c**) samples with 3% carbon (non-ionic: ● BG; ● CG; ○ APG; ○ LQ; anionic: ■ Tego; ■ H66).

To understand the impact of the surfactant on the foam formation based on several types of carbon, Figure 2 shows the Δh variation function of the pore size (as determined from the software “ImageJ” [27]) for the three types of carbon (3 wt.%) with 0.1 wt.% of various surfactants. For the biochar carbon (Figure 2a), we observe an increase in the pore size with the volume expansion. Globally, the nonionic surfactants display a fast increase to reach the maximum with BG (Γv = 1.6 mm and Δh = 38%). The CG surfactant has the lowest pore size and lowest volume expansion of approximately 0.4 mm and 9%, respectively. For anionic surfactants, the pore size remains weak at 0.4 mm (Δh = 6%) and 0.6 mm (Δh = 18%) for H66 and Tego, respectively. Graphite 75 and 99 also induce the same behavior for nonionic surfactants (Figure 2b,c). The BG surfactant shows the highest pore size of approximately 1.7 mm for a volume expansion of 40%, whereas the CG surfactant induces the lowest pore size of approximately 0.9 mm for a volume expansion of 17%. This can be explained by an open macro porous network leading to porosity [28]. The anionic surfactants used lead to a decrease of Γv with volume expansion in agreement with the CMC value, which was exceeded. Tego 653 induces a pore size of 0.4 mm for a volume expansion of 19%, whereas the H66 surfactant induces a pore size of 0.9 mm for a volume expansion of 10% (Figure 2c). To explain this behavior, it should be pointed out that anionic surfactants have electrostatic charges that can interact with the alkaline cations in the reaction mixture, thus limiting pore coalescence, as evidenced by Ping et al. [17]. Conversely, non-ionic surfactants will promote the coalescence of bubbles during the formation, and thus higher pore sizes [29]. Regardless of the type of carbon, the particle size is between 40 and 120 µm. The carbon content introduced is kept constant to avoid any changes. Nevertheless, biochar contains some organic compounds, graphite 75 contains fly ash, and graphite 99 is the purest. In the presence of an anionic surfactant, regardless of the type of carbon, the surface concentration corresponds to a closely packed monolayer of molecules oriented parallel to the surface [30], which are responsible for steric hindrance favoring the pore size. In the presence of the anionic surfactant, the decrease in pore size can be explained by the effect of only stripe patterns at the surface, which minimizes the bubbles and, consequently, the pore size [31]. The pore size and volume expansion values are presented in Table 1. In particular, for a percentage of 0.1%, the expansion values and pore size were similar for BG and GG^75^ at approximately 38% and 1.6 mm, respectively, and at 25% and 2.5 mm for GG^99^. This can be explained by the powder purity. In the presence of a synthetic powder, such as GG^99^, geopolymerization reactions are not modified; however, with impurities from the secondary reactions that occur, there is a limitation of expansion [32].

The internal morphologies of the samples based on biochar and various types of surfactant are presented in Table 2. All samples exhibit a gray color owing to the black color of carbon and spherical closed pores. Small differences can be observed in the function of carbon nature. However, the main difference is the texture of the samples, which seems to be controlled by the surfactant type. In fact, using the BG surfactant, the samples are foam and more pores are observed, owing to the high volume expansion induced by this type of surfactant at 0.1 wt.% addition. All other types of surfactants for an addition of 0.1% induced dense samples for all types of carbon used.

**Figure 3:**
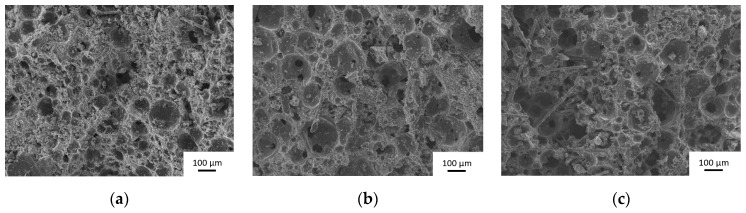
SEM micrographs of (**a**) GB, (**b**) GG^75^, and (**c**) GG^99^ samples.

In order to more precisely investigate the microstructure, an example of SEM images obtained for GB, GG^75^, and GG^99^ samples using 0.1 wt.% of BG surfactant is given in Figure 3.

Whatever the sample, a porous structure distributed homogeneously throughout the matrix is noticed. The tubular microstructure is attributed to basalt fibers added in all samples. GB samples show small spherical pores. More coalesce of pores is observed in the case of GG^75^ and especially for the GG^99^ sample. Consequently, small differences can be detected in the function of carbon type. However, the type of surfactant is responsible for the obtained microstructure.

### 2.2. Dielectric Properties

**Figure 4:**
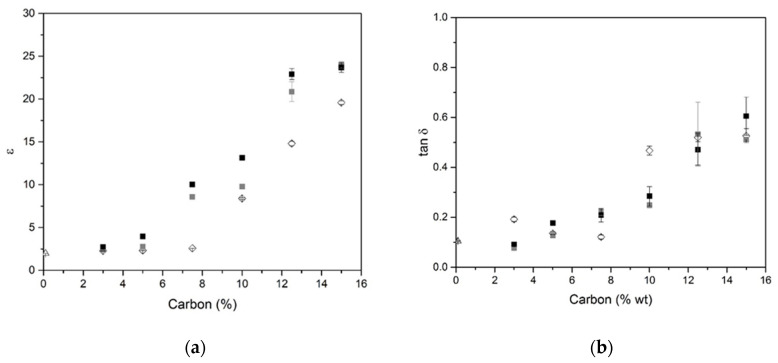

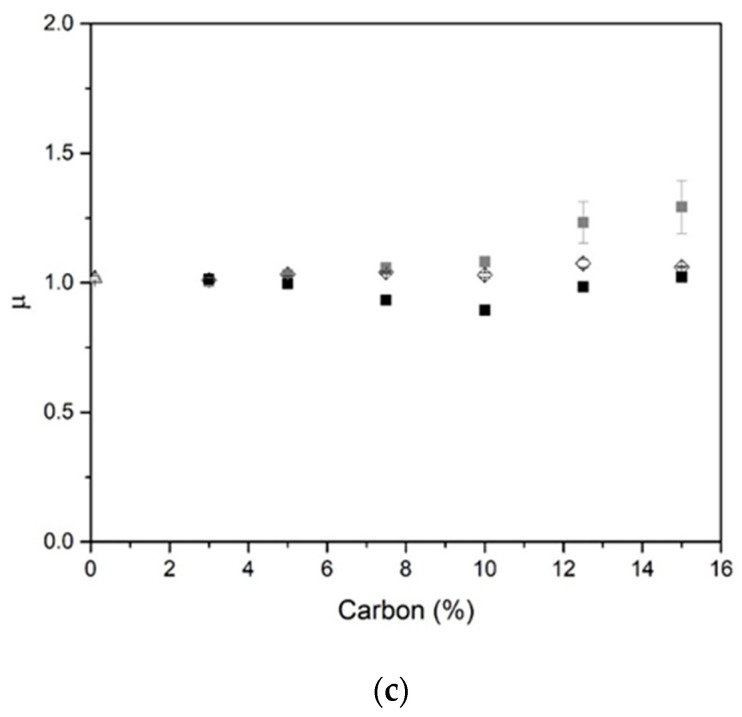
Values of ε (**a**), tan δ (**b**), and µ (**c**) at 2.45 GHz in function carbon content for GB (◊), GG^75^ (■), and GG^99^ (■) samples based on 0.1 wt.% BG (kept at 20 °C and 60% humidity—laboratory conditions).

The previous data highlight that the BG is the surfactant that provides the best expansion; consequently, it is retained with 0.1 wt.% addition. Several samples based on this content with three different types of carbon (biochar, graphite 75, and graphite 99) were synthesized, and their dielectric properties were investigated (ε (a), tan δ (b), and µ (c) at 2.45 GHz) (Figure 4). All samples present similar behaviors, increasing carbon contents, and induce an increase of ε values (Figure 4a). Samples without carbon addition (G) present an ε value of 1.987; addition of 3% of carbon induces an increase of the ε value to 2.26 for biochar. The ε increases slowly up to a rate of 7%, and beyond that, there is a sharp increase to a value of 18.35 for 15% biochar. This can be explained by the percolation rate being much higher than the conduction threshold, in accordance with percolation theory [33]. The same tendency is observed as for ε for the dielectric loss function of carbon content for samples based on biochar, graphite 75, and graphite 99 (Figure 4b). Essentially, the formation of macro pore channels leads to an apparent enhancement of dielectric properties in agreement with the porosity [34]. The value of tan δ for samples without carbon insertion was approximately 0.1. Upon increasing the carbon content, the tan δ values increase for all compositions. For example, for samples based on biochar, the values varied between 0.19 and 0.45 for additions between 3 and 15%. All compositions (Figure 4c) showed similar values of µ, approximately 1, owing to the diamagnetic property of carbon [35]. The same behavior was reported in the literature for composites based on graphite; when graphite content is increased, the dielectric constants of the composite increased gradually, whereas the magnetic constants stayed almost unchanged, indicating that dielectric losses are the main microwave-absorbing mechanism of the composites [36]. The dielectric data as a function of humidity are provided in Table 3.

**Figure 5:**
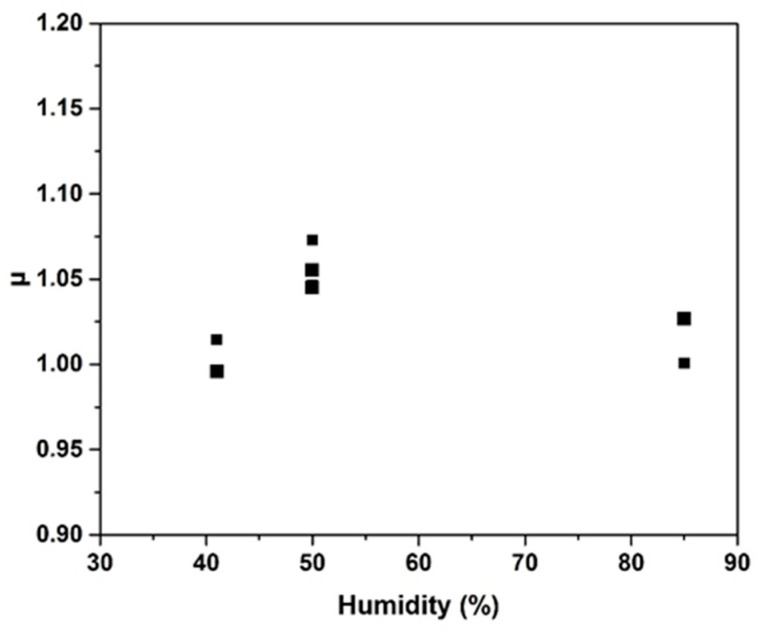
Values of µ at 2.45 GHz function of humidity for GG^99^ (▪) and GG^99^F1 (■) samples kept at a controlled humidity by climatic chamber.

The samples were kept at 20 °C at 50% relative humidity (RH) for 12 days; then, they were maintained at 20 °C at 85% RH for 4 days and returned to 50% HR for 3 days. The increase of humidity induced an ε increase of 150% regardless of the sample. For the samples based on biochar, the initial ε was 2.26 at 40% humidity. Increasing the humidity to 50% and using a climatic chamber to control the humidity, the ε values increased to 2.7; at a humidity of 85%, the ε increased to 4.27. Upon setting the humidity to 50% RH, the values remained approximately the same as the initial values. The same tendency was observed for tan δ. The same behavior was reported in the literature for polymeric composites; upon increasing the humidity from 30% to 90%, the dielectric values increased [36]. Humidity affects the permittivity and the loss tangent, and an increase is observed, as well as an increase in humidity; however, the values decreased with humidity. This behavior has been previously observed and was attributed to water and cation mobility in the geopolymer [37]. The values of µ remained at approximately 1 for all compositions owing to the diamagnetic properties of carbon. Humidity did not affect the magnetic properties. The same behavior was observed in our previous study on a dense geopolymer with magnetite addition [37]. To ensure the accuracy of the magnetic data, 1% addition of magnetite (particle size 44 µm) was added to the GG^99^ sample to investigate the effect of magnetite and humidity on the composition. Figure 5 presents the evolution of the µ values at different humidity (40, 50, and 85%) for both samples (GG^99^ and GG^99^F1). The µ values for both samples were close to 1 for all humidities owing to the low concentration of magnetite used (1%). The small difference between the µ values upon magnetite addition was in accordance with the values of permeability found in prior studies for geopolymer composites [38]. Magnetite addition induced a slight increase in permeability values owing to the magnetic properties of magnetite. The permeability of magnetite powder for a particle size between 28 and 63 µm at 25 °C and a frequency of 2.45 GHz is between 2 and 3 according to the literature [39,40]. The increase in water content after humidity exposure can be explained by the increase in free and physiosorbed water. This type of water favors the mobility of free K^+^ and increases the electrical conductivity of the geopolymer [41]. This suggests a modification of the dielectric properties under a relative humidity change. Humidity affects the dielectric properties (permittivity and tan δ), but has a slight effect on the permeability. Some recent studies have shown a relationship between humidity and permeability. For example, Cerovic et al. [42] have shown an increase of permeability with the increase of relative humidity, which is most intense with highly moisture-dependent materials such as cotton. They explain that this is because water molecules enhance electric polarization. Li et al. [43] have demonstrated that different moisture amounts inside concrete may affect its relative permeability, because water is a weak magnetic medium that exists in concrete in the form of a pore solution.

## 3. Discussion

**Figure 6:**
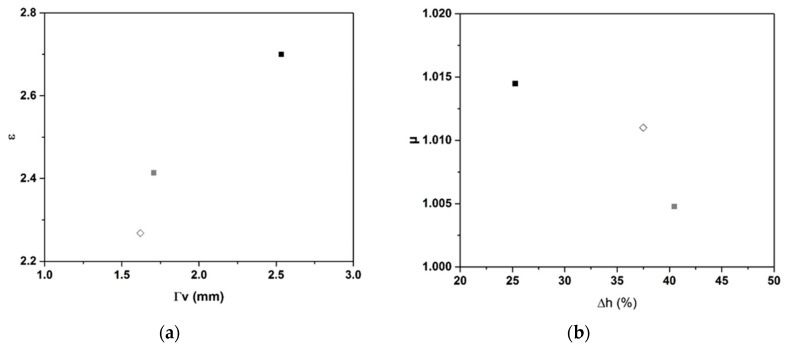
Values of ε at 2.45 GHz function of pore size (**a**) and values of µ at 2.45 GHz function of volume expansion (**b**) for GB (◊), GG^75^ (■), and GG^99^ (■) samples with 3% carbon content and 0.1% BG surfactant.

To understand the changes in ε and tan δ as a function of each type of carbon, we plotted Figure 6 to show their changes as a function of the porosity data (size pore and volume expansion). The ε value increased with the pore size (Figure 6a). Samples based on biochar presented an ε value of 2.27 for a pore size of 1.6 mm, graphite 75-based samples presented values of ε of 2.41 and a pore size of 1.7 mm, and graphite 99-based samples presented an ε value of 2.7 and a pore size of 2.53 mm. The higher pore size in the case of graphite 99 is owing to its crystallinity and the purity of this carbon hierarchical structure [43], as can be observed on the X-ray diffraction (XRD) pattern. In graphite 75-based samples, the fly ash may participate in the geopolymerization reaction, which strengthens the foam skeleton and limits the coalescence of pores; therefore, the pore size decreases. Indeed, the chemical elements Al and Si contained in the fly ash could participate in the polycondensation reaction to modify the geopolymer network and reinforce the solid skeleton. Furthermore, it was found in the literature that, for alkali-activated materials based on fly ash, the porosity decreased with the increase in fly-ash content, inducing an increase in the dielectric values. The porosity was inversely proportional to the dielectric value, meaning that, when the porosity increases, the values of ε decrease [44]. Biochar was also proven to participate in the geopolymerisation reaction [32], leading to a lower pore size. Previous work of Farges et al. [32] has demonstrated that geopolymers based on biochar can be obtained. Several type of geopolymers such as foam or dense were synthetized with different working properties. Furthermore, most organic compounds have only a small or modest impact on the permittivity, whereas inorganic fillers are very effective with a high dielectric constant, such as carbon black and TiO_2_. This is owing to the dipole moments in the repeat units, which do not balance each other. Consequently, the presence of some impurities, such as organic compounds or fly ash, in the carbon content could affect the dielectric properties. The dielectric constant is also affected by the free volume, which is the volume that is not occupied by the molecules or repeat units. A decrease in the dielectric constant induces a decrease in polarizable groups per unit volume owing to crystallization, which increases the free volume [45]. The same occurs for pores, which are filled with air whose relative permittivity is approximately one [45].

The µ values decreased with an increase in volume expansion (Figure 6b). Samples based on biochar had the highest volume expansion, whereas for graphite, increasing the carbon content induced a decrease in the volume expansion. The permeability of geopolymers based on various types of carbon (graphite or carbon fibers) typically fluctuates around 1 to 1.1 [38,46]. Different amounts of magnetic impurities can be found in the carbon precursor that can induce intrinsic magnetism in these samples. If some finely dispersed impurity was present in the starting powder, it could undergo clusterization. Some sharp changes in the magnetic properties could result from such a process [47]. For fly ash, increasing its content was found to induce an increase in the magnetic properties owing to the higher iron content and presence of more magnetic minerals [48]. Indeed, if there is magnetite addition, the values are supposed to increase when holding the value of magnetite at 2.5 [39,40]. None of the magnetic properties were found to vary consistently with volume expansion, although changes in magnetic properties with porosity could be detected using magnetic measurements [49]. Consequently, the nature of carbon (purity and crystallinity) influences in a major part the dielectric properties and seems to have little effect on the magnetic one. Indeed, low ε values can be obtained for low pore size samples and with impure carbon.

## 4. Materials and Methods

### 4.1. Materials and Synthesis

**Figure 7:**
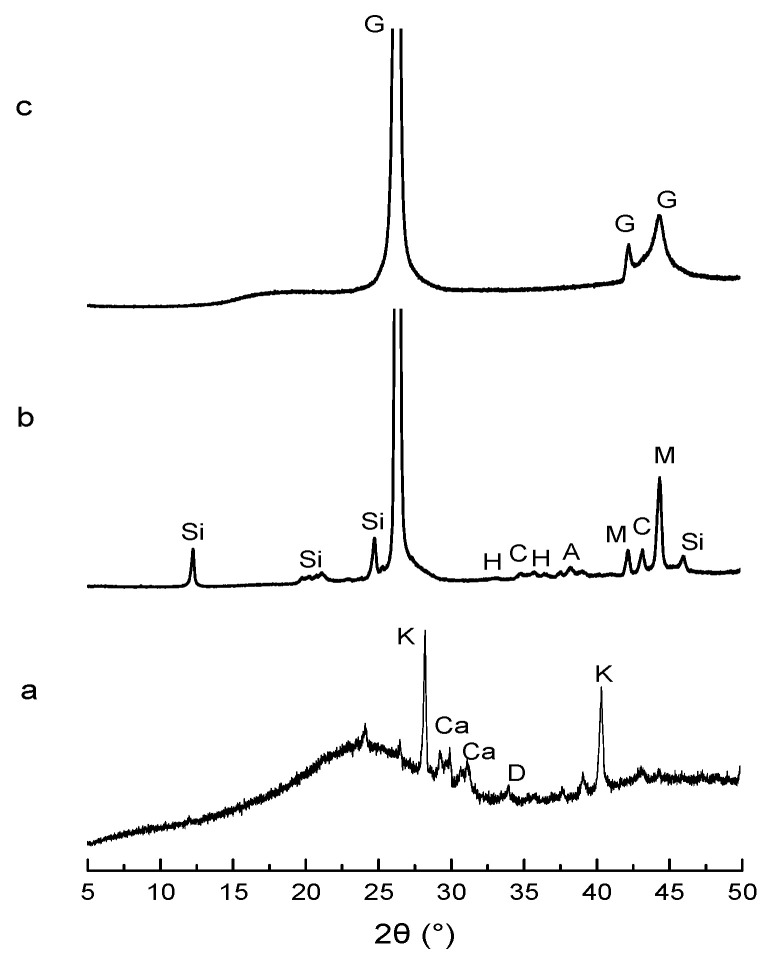
X-ray diffraction (XRD) patterns of (**a**) biochar, (**b**) G75, and (**c**) G99 (where G—graphite-2H 00-041-1487, Si—SiO2 00-061-0035, H—hematite 00-033-0664, C—corundum 04-004-5434, A—aluminium magnesium 04-007-1292, M—magnetite 01-074-1909, K—KCl 01-076-3384, Ca—calcite 00-066-0867, D—dolomite 01-083-5726).

The materials used for the synthesis of the geopolymer samples were silicate solution S1 with Si/K molar ratios of 0.54 and M4 metakaolin with a Si/Al molar ratio of 1 [14]. To obtain a material with dielectric properties, three types of carbon were used with different carbon contents, biochar (81% of carbon) and two types of graphite with 75 wt.% and 99 wt.% of carbon (Table 4). Various percentages of carbon were used: 3, 5, 7.5, 10, 12.5, and 15 wt.%. XRD patterns (Figure 7) confirm the purity of graphite 99 wt.%. For graphite with 75 wt.%, impurities detected are magnetite, hematite, corundum, and aluminium magnesium alloy, which are attributable to fly ash. Biochar pattern exhibits a broad dome centered at about 22.5°, characteristic of the amorphous structure in addition to crystalline phases such as sylvite, calcite, and dolomite.

Various types of surfactants were used to investigate the influence on the volume expansion (Table 5). Basalt fibers (length of 3 mm) were used to reinforce the matrix for mechanical properties.

**Figure 8:**
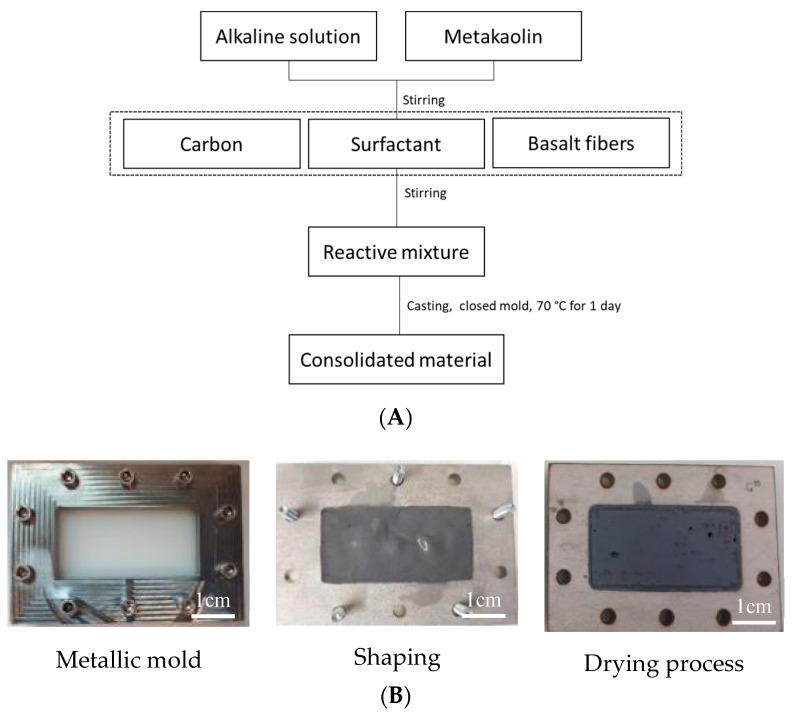
(**A**) Synthesis protocol of geopolymer samples and (**B**) shaping for dielectric measurements for samples.

Figure 8A shows the synthesis protocol for the geopolymer samples. The silicate solution was combined with metakaolin, basalt fibers, and surfactants with different quantities and types of carbon. The obtained mixtures (Si/Al molar ratio is 1.4, Si/K molar ratio is 1.79, and Si/C molar ratio is 1) were placed in a closed mold at 70 °C for 1 day. Samples (86 × 43 × 10 mm) for the dielectric measurements were made, as presented in Figure 8B.

**Figure 9:**
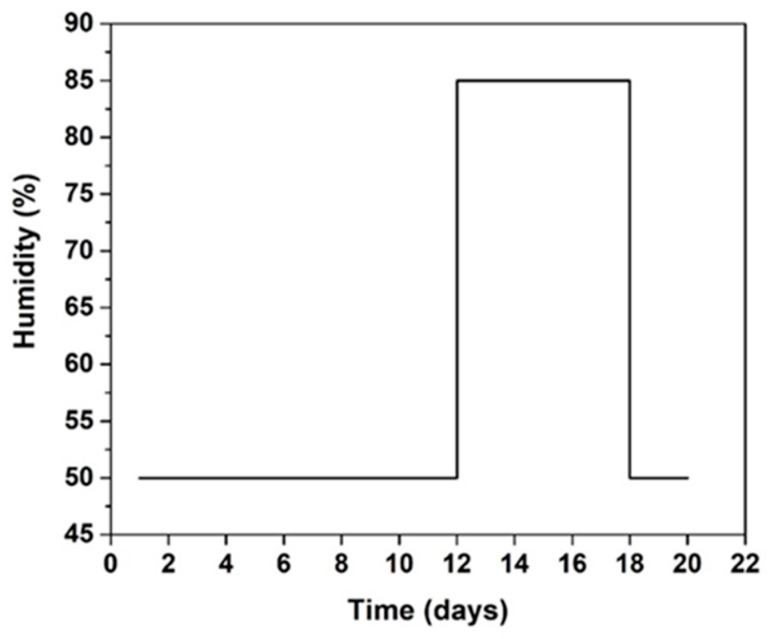
Example of cycle of humidity treatment.

The metallic mold was fixed using screws and screw nuts between the polyethylene plates. The reactive mixture was casted in the metallic mold and covered. Samples were kept at 70 °C for 1 day and afterwards removed from the plates and dried in a vertical position for 1 day. After the drying process, the samples presented no cracks, but some pores were observed on the surface. To investigate the influence of humidity, samples were kept at a controlled humidity. The cycle of humidity treatment is presented in Figure 9. During the first days, samples were kept at 20 °C at 50% humidity, and then at 85% humidity for 5 days; then, the humidity was changed back to 50% for 3 more days. The nomenclature and compositions of the samples are presented in Table 4. Samples were identified as GCx, where G represents the reference composition, C represents the type of carbon (B—biochar, G^75^ and G^99^—the two types of graphite with 75 and 99% of carbon), and x is the percentage of carbon used (3, 5, 7.5, 10, 12.5, or 15%) in the composition.

### 4.2. Technical Characterization

The volume expansion was calculated using Equation (1):

(1) $$\Delta h=\frac{\left(h_{f}-h_{i}\right)}{h_{i}}\times 100$$

where ∆h—volume expansion (%); h_i_—initial height before thermal treatment (mm); h_f_—final height after thermal treatment (mm).

X-ray diffraction patterns were acquired via X-ray diffraction (XRD) experiments on a Bruker-D8 Advance diffractometer Bragg Brentano powder diffractometer using CuKα radiation (λKα = 0.154186 nm). The analytical range is between 5° and 50° (2θ) with a step of 0.015°/s.

The morphology of the material was observed using JOEL IT300. A carbon fine layer was deposited on the samples before the observations.

The pore size distribution is established from analyses of different sections of the sample at different heights using the Image J software [27]. The pore diameters are counted according to their size in order to determine the value of the average diameter Γ_v_ [50,51] of each cut calculated using Equation (2) [52]:

(2) $$\Gamma_{v}=\frac{\sum_{i=0}^{n}n_{i}d_{i}^{4}}{\sum_{i=0}^{n}n_{i}d_{i}^{3}}$$

where Γ_v_—average diameter (mm); d_i_—the pores diameter of class i; n_i_/n—the ratio of the number of pores of class i to the total number of pores.

**Figure 10:**
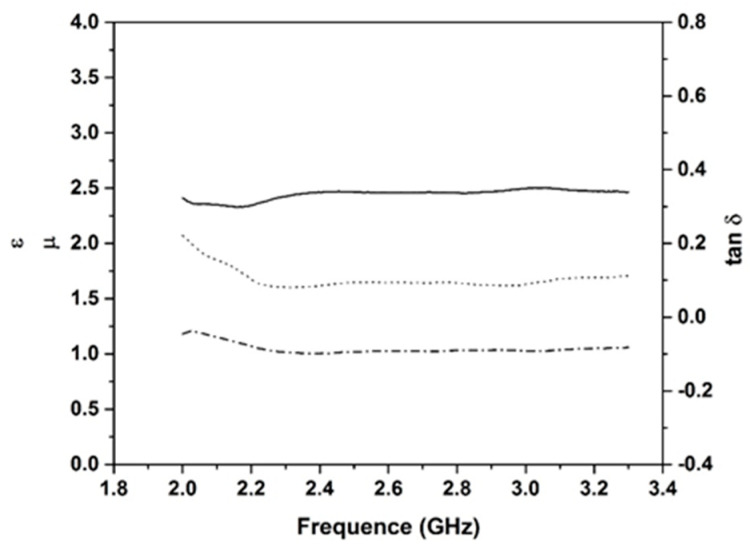
Example of dielectric measurements results of the GB sample (- ε; -·-·- µ; - - - tan δ).

Dielectric measurements were carried out using a Vector Network Analyzer (VNA) Keysight E5063A, which provided high-precision scattering parameters (S_ij_) from which the dielectric and magnetic parameter values were extracted (permittivity, permeability, and loss tangent). For more homogeneous measurements and characterization of the samples, the loaded rectangular waveguide method was chosen despite its reduced frequency band in the mono mode operation (fc_TE10_ = 1.735 GHz and fc_TE20_ = 3.47 GHz for the WR340 standard used in this study). Measurements of standard samples (Plexiglas and Teflon) were made, and comparisons of complex permittivity extracted by the Nicolson-Ross-Weir (NRW) algorithm [53,54] and by another free space method [55] showed good agreements and validated the entire process. The sample was connected by a phase- and amplitude-stable cable to a VNA, which was then calibrated by Thru, Reflect, Line (TRL) calibration technic with WR340 standards. Figure 10 shows an example of dielectric measurements of the GB_3_ samples between 2 and 3.3 GHz. Dielectric measurements were performed to determine the values of the real part of the dielectric constant, which is known as the real part of the relative permittivity (ε’_r_) and indicates the ability of the material to store microwave energy, tan δ_ε_ and tan δ_µ_ (not taking account diamagnetic materials), which are the dielectric and magnetic losses that quantify a dielectric material’s inherent dissipation of electromagnetic energy. The values of ε, for frequencies between 2 and 3.3 GHz, for this composition were approximately 2.5, whereas tan δ values were approximately 0.1.

## 5. Conclusions

The objective of this work is the synthesis route of a new eco-friendly geopolymer for applications as absorbent material with various compositions and additives. The effects of the formulation based on various type of carbon, surfactant, and magnetite were investigated.

- Surfactant addition induces the volume expansion owing to a change in interfacial strength. However, the nonionic surfactant was preferred over the anionic surfactant, thanks to its performance on the volume expansion at lower concentrations.
- Dielectric investigations reveal an increase of permittivity with increasing carbon content, for example, ε = 2.27 and tan δ value of 0.19.
- The addition of magnetite reveals only a minor impact on the samples magnetic properties.
- An increase is observed with increasing humidity, but a reversible behavior is observed when the humidity is decreased.

## Figures and Tables

**Table 1:** Volume expansion and pore size function of percentage of various type of surfactant for biochar and graphite based geopolymers.

Type of Surfactant	Percentage of Surfactant	Volume Expansion (%)			Pore Size (mm)		
		Biochar	Graphite 75%	Graphite 99%	Biochar	Graphite 75%	Graphite 99%
BG	0.1	38	40	25	1.6	1.7	2.5
CG	0.1	9	18	7	0.4	0.9	0.6
APG	0.1	11	19	16	1.0	1.3	1.7
LQ	0.1	9	18	9	0.5	1.0	1.0
Tego	0.1	19	25	19	0.6	0.6	0.4
H66	0.1	6	11	10	0.4	1.2	0.9

**Table 2:** Visual aspect and internal morphology of samples based on various type of carbon with 0.1% addition of different types of surfactants.

Name of Surfactant	Internal Morphology		
	Biochar	Graphite 75	Graphite 99
BG	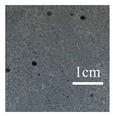	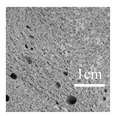	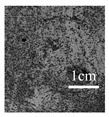
CG	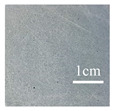	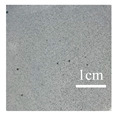	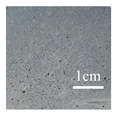
Tego	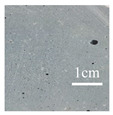	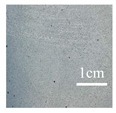	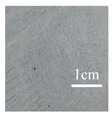
H66	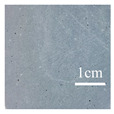	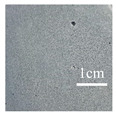	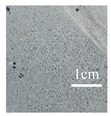

**Table 3:** Values of ε, tan δ, and µ for the various samples at 2.45 GHz, 20 °C, and different humidity.

Type of Carbon	Humidity (%)								
	50 (during 12 days)			85 (4 days)			50 (3 days)		
	ε	tan δ	µ	ε	tan δ	µ	ε	tan δ	µ
Biochar	2.7	0.13	1.04	4.27	0.37	1.04	2.75	0.16	1.09
Graphite 75	2.47	0.09	1.00	3.82	0.28	0.98	2.50	0.11	1.03
Graphite 99	2.87	0.12	1.01	4.76	0.35	1.00	2.95	0.15	1.07

**Table 4:** Carbon type details.

Carbon Type	Supplier	Carbon (%)	Particle Size (µm)	Impurities (%)
Biochar	Maillot	81	4–119	19
Graphite 75	Alfa Aesar	75	45	25
Graphite 99		99	45	1

**Table 5:** Surfactants details.

Name of Surfactant	Supplier	Type	pH	Density (g/cm^3^)	CMC (ppm at 25 °C)
TRITON™ BG-10 (BG)	Dow	nonionic	7.6	1.152	1591
TRITON™ CG-110 (CG)		nonionic	5.7	1.150	1748
CAFLON APG C6 SMP (APG)	Univar B.V.	nonionic	7–9	1.150–1.170	*
SPAN™ 80-LQ-(RB) (LQ)	Croda	nonionic	*	*	*
TEGO^®^ Dispers 653 (Tego)	Evonik	anionic	8–9	1.075	*
TRITON™ H-66 (H66)	Dow	anionic	8.4	1.249	*

* data not provided.
